# Pricing Strategies to Encourage Availability, Purchase, and Consumption of Healthy Foods and Beverages: A Systematic Review

**DOI:** 10.5888/pcd14.170213

**Published:** 2017-11-02

**Authors:** Joel Gittelsohn, Angela Cristina Bizzotto Trude, Hyunju Kim

**Affiliations:** 1 Johns Hopkins Bloomberg School of Public Health, Department of International Health, Global Obesity Prevention Center and Center for Human Nutrition, Baltimore, Maryland

## Abstract

**Introduction:**

Food pricing policies to promote healthy diets, such as taxes, price manipulations, and food subsidies, have been tested in different settings. However, little consensus exists about the effect of these policies on the availability of healthy and unhealthy foods, on what foods consumers buy, or on the impact of food purchases on consumer health outcomes. We conducted a systematic review of studies of the effect of food-pricing interventions on retail sales and on consumer purchasing and consumption of healthy foods and beverages.

**Methods:**

We used MEDLINE, Embase, PsycINFO, Web of Science, ClinicalTrials.gov, and the Cochrane Library to conduct a systematic search for peer-reviewed articles related to studies of food pricing policies. We selected articles that were published in English from January 2000 through December 2016 on the following types of studies: 1) real-world experimental studies (randomized controlled trials, quasi-experimental studies, and natural experiments); 2) population studies of people or retail stores in middle-income and high-income countries; 3) pricing interventions alone or in combination with other strategies (price promotions, coupons, taxes, or cash-back rebates), excluding studies of vending-machine or online sales; and 4) outcomes studies at the retail (stocking, sales) and consumer (purchasing, consumption) levels. We selected 65 articles representing 30 studies for review.

**Results:**

Sixteen pricing intervention studies that sought to improve access to healthy food and beverage options reported increased stocking and sales of promoted food items. Most studies (n = 23) reported improvement in the purchasing and consumption of healthy foods or beverages or decreased purchasing and consumption of unhealthy foods or beverages. Most studies assessed promotions of fresh fruits and vegetables (n = 20); however, these foods may be hard to source, have high perishability, and raise concerns about safety and handling. Few of the pricing studies we reviewed discouraged purchasing and consumption of unhealthy foods (n = 6). Many studies we reviewed had limitations, including lack of formative research, process evaluation, or psychosocial and health assessments of the intervention’s impact; short intervention duration; or no assessment of food substitutions or the effects of pricing interventions on food purchasing and diets.

**Conclusion:**

Pricing interventions generally increased stocking, sales, purchasing, and consumption of promoted foods and beverages. Additional studies are needed to differentiate the potential impact of selected pricing strategies and policies over others.

## Introduction

Pricing strategies to encourage the availability, purchasing, and consumption of healthy foods and beverages have received increased attention in the past decade, in the United States and worldwide. Various pricing strategies have been studied in different settings, including taxes and price manipulations of sugar-sweetened beverages (SSBs), high calorie–low nutrient foods or foods high in added sugars or saturated fats, and subsidies of fruits and vegetables. Despite these studies, little consensus exists about the effectiveness of these pricing strategies in changing the availability and affordability of healthy and unhealthy foods or their effect on consumer outcomes (ie, foods purchased, foods consumed, and health). Furthermore, little consensus exists about how pricing strategies function, alone or combined with health behavior interventions or as part of multi-level interventions.

Reviews were conducted previously on related topics. Nine recent reviews (from 2010 through 2015) examined the effect of taxes, subsidies, or their pooled effect on food consumption, consumer purchases, body weight, or diet-related chronic diseases (1–9). However, many of these reviews described laboratory-based or simulation studies (6–8). Only one systematic review described field intervention studies (9) and focused on subsidies to increase purchasing of healthy foods. Few focused on implementation and outcomes of pricing interventions at both the supply (retail) and demand (consumer) levels in actual communities.

Decision makers need a systematic review of the effectiveness of pricing incentives and disincentive strategies on availability, purchasing, and consumption of healthy and unhealthy foods and beverages at the consumer and retail levels. Therefore, our goal was to answer the following questions: 1) How do pricing incentives and disincentives influence access, purchasing, and consumption of healthy and unhealthy foods and beverages among various populations in high-income and middle-income countries? 2) What additional work is needed to enable communities, states, and countries to identify the best combination of strategies?

## Methods

### Data sources

We conducted a systematic review of English-language, peer-reviewed articles describing studies that evaluated the effectiveness of pricing incentives and disincentive strategies on purchasing and consumption of healthy and unhealthy foods and beverages in high-income and middle-income countries in various socioeconomic settings. We searched 6 electronic databases — MEDLINE, Embase, PsycINFO, Web of Science, ClinicalTrials.gov, and the Cochrane Library — from January 2000 through December 2016 for relevant studies.

We developed a search strategy based on medical subject heading (MeSH) terms and based on the text and key words of key articles we identified a priori (Appendix). We used Boolean operators to combine keywords and MeSH terms for a focused search. We developed 3 topics based on our research question (incentive/disincentive, food intake, and food purchasing), and we then included key words and MeSH terms representing each term. Search terms were *pricing strategies, incentive, reimbursement, commerce, disincentive, reward, taxes, monetary incentive, consumer behavior, marketing, cost savings, food purchasing, food supply, dietary intake, eating behavior, food intake, food and beverages,* and *snacks*.

### Study selection

We selected the following types of human studies published in English in peer-reviewed journals, from 2000 through 2016: 1) experimental studies (randomized controlled trials, quasi-experimental studies, and natural experiments, excluding reviews and cross-sectional, qualitative, and simulation models studies); 2) population studies of people or stores in middle-income and high-income countries; 3) studies of pricing interventions conducted alone or in combination with other strategies (price promotions, coupons, taxes, or cash-back rebates), excluding studies of vending-machine or online sales; and 4) outcomes studied at the retail (stocking, sales) or consumer (purchasing, consumption) levels. Additional criteria were that study outcomes were assessed at the retail level (stocking, sales) or consumer level (purchasing, consumption) and that the study was not an evaluation of a government program in schools (eg, a school-based food assistance program).

Two reviewers (A.C.B.T., H.K.) reviewed abstracts and full articles independently to assess eligibility for inclusion. H.K. confirmed or corrected A.C.B.T.’s data abstractions for completeness and accuracy. We also conducted a reference list search on the studies we selected for review and identified 5 eligible studies. Lastly, we identified all peer-reviewed publications associated with each study and cited only those that contributed to this review.

### Data extraction

For the synthesis, we employed an adjudication approach. We used a series of descriptive criteria to characterize each study: project name, target population, model or theory, study goal, foods and beverages that were the intervention’s focus and its retail venue, sample size, intervention strategies, study design, study duration, formative research, feasibility assessment, process evaluation, impact measures and results, sustainability, quality of research, study limitations, and study recommendations (Appendix Table 1). Two reviewers (A.C.B.T., H.K.) analyzed each study independently and provided a long and a short response for the descriptive criteria. A third reviewer (J.G.) reviewed the descriptions and agreed or disagreed to the study’s inclusion. Where there was disagreement, the third reviewer broke the tie.

We organized data into the following categories: 1) a description of each study; 2) a description of the intervention, pricing strategies, and the study evaluation; and 3) main results and study implications. Data were then grouped by type of pricing intervention categories: 1) financial discount on healthy foods and beverages, 2) redeemable coupons or vouchers for healthy foods and beverages targeting participants in food assistance programs, 3) redeemable coupons or vouchers for healthy foods and beverages targeting consumers not participating in food assistance programs, 4) cash rebates, and 5) disincentive strategies for unhealthy food and beverage purchases (eg, tax, alone or combined with a strategy promoting healthy foods).

## Results

Searches returned 2,076 articles, and 1,677 were screened after excluding duplicates (ie, the same article in different research databases) and by refining the searches by year, language, and species. After elimination of 1,625 for not meeting our study criteria, 52 were fully assessed for eligibility; 27 were excluded and 5 were included after a reference list search. Thus, 30 distinct studies in 63 articles were included in the final analysis (Figure). The number of peer-reviewed publications per study varied from 1 to 7, with a median of 2 per study.

**Figure:**
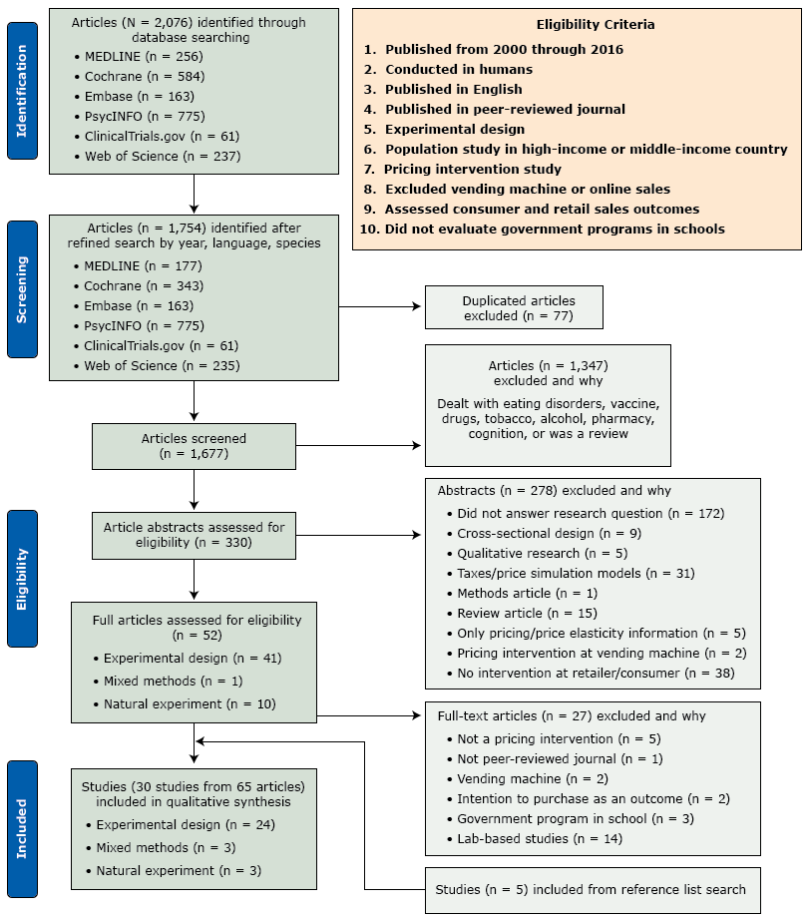
Selection process, systematic review of pricing strategies to encourage purchasing and consumption of healthy foods and beverages, 2000–2016.

### Description of studies

The 30 studies included in the review were conducted in 9 countries: the United States (n = 17), Australia (n = 2), New Zealand (n = 2), France (n = 2), Canada (n = 1), the United Kingdom (n = 1), South Africa (n = 1), Denmark (n = 1), Belgium (n = 1), Peru (n = 1), and Mexico (n = 1) (Table 1). The largest number of studies (n = 8) took place in the northeastern United States (Table 1). Most studies (n = 18) did not report the use of a theoretical model. Of the 12 that did, social cognitive theory was most commonly mentioned (n = 5), followed by the social ecological model (n = 5). The most common study design (n = 15) was a randomized controlled trial, with randomization either at the group or individual level.

**Table 1:** Studies of Pricing Strategies to Encourage Purchasing and Consumption of Healthy Foods and Beverages, 2000–2016

Type of Study/Study Name	Design	Sample Size	Duration	Target Group/Dates	Model/ Theory	Goal or Purpose
**Financial discounts on healthy foods and beverages**						
Baltimore Healthy Carryouts (10–17)	Quasi-experimental, 2 arms	8 Carryout restaurants, 186 consumers	8 months	Low-income black residents in Baltimore, Maryland, 2011	SCT, SEM, SM	To improve healthy food purchasing in carryout restaurants offering a reduced-price combination meal; to increase total sales of healthy foods and carryout revenue
B’More Healthy Retail Rewards (18,19)	RCT, 4 arms: 1) pricing incentive at wholesaler, 2) communications, 3) pricing and communications, 4) control	24 Corner stores, 1 wholesaler, 360 store customers	6 months	Low-income black adult consumers in Baltimore, Maryland, 2012–2013	SCT, SEM	To assess the impact of separate and combined pricing and communication strategies on food purchasing and on retailer stocking and sales
Not named (healthy foods at swimming pools) (20)	Quasi-experimental successive and additive interventions: signage, taste-testing, price reduction; qualitative and quantitative observations	2 Concession stands, 650 adults, 342 children	5 months	Pool patrons: children and adults living in Alberta, Canada, in 2012	BE	To assess the comparative and additive efficacy of 2 nudges and an economic incentive in supporting healthy food purchases
HealthWorks (21–24)	Group-randomized controlled trial, 2 arms: 1) intervention, 2) control	6 Worksites, 1,672 adults	2 years	Employees at 6 worksites in Minneapolis–St Paul, Minnesota, 2006–2008	SCT	To positively influence weight gain prevention
Not named (Mississippi Healthy Beverages) (25)	Quasi-experimental design: first year, no intervention; second year, intervention	15 Schools (no individual-level data)	2 years	School-aged children (K–12), various income levels, living in Mississippi, 2005–2006	None reported	To improve purchase of healthy beverage choices; maintaining profit in school stores by increasing availability, reducing prices, and labeling
Not named (multi-component intervention in sports clubs) (26)	Group-randomized controlled trial, 2 arms: 1) intervention, 2) control	85 Clubs, 1,394 club members	2.5 years	Adult sports club members in New South Wales, Australia, 2009–2012	SEM	To increase consumption, availability, and sales of non-SSBs and FV in sporting club canteens
Supermarket Healthy Eating for Life (SHELf) trial (27–30)	RCT, 4 arms: 1) skill-building, 2) price reduction, 3) skill-building and price reduction, 4) control	642 Women; impact data reported on 574 women	3 months	Female, main household food shoppers, low-SES and high-SES neighborhoods in Australia, 2011–2012	SEM, SCT	To increase purchasing and consumption of FV, reduce purchasing of SSBs, increase purchasing of low-calorie soft drinks and water
Supermarket Healthy Options Project (SHOP) (31–34)	RCT, 4 arms: 1) tailored nutrition education, 2) price reduction, 3) combination of tailored nutrition and price reduction, 4) control	1,104 Adults	6 months	Diverse adult food shoppers, including Maori/Pacific Islanders in New Zealand, 2007–2009	None reported	To test the effect of price discounts and nutrition education on supermarket food and nutrient purchases
Not named (Lima University cafeteria study) (35)	Quasi-experimental, 3 phases: 1) location only, 2) location and signage, 3) location, signage, and price reduction	150 Students; qualitative interviews, 12 students	6 weeks	Young adults, college students in Lima Peru, 2016	SM	To improve fruit purchases in a university cafeteria
**Redeemable coupons or vouchers for healthy foods and beverages targeting participants in food assistance programs**						
Farmers Market Fresh Fund Incentive Program (36)	Mixed-methods, pre–post repeated measure design; no comparison group	908 Participants; 252 with longer follow-up (1 y)	19 months	Low-income urban Hispanic families in San Diego, California, 2010–2011	None reported	To examine the effect of a doubling incentive on number of farmers market visits, consumer diets, and economic benefits to farmers
Project FRESH (Farm Resources Encouraging and Supporting Health) (37)	Quasi-experimental, 4 arms: 1) coupon, 2) education, 3) coupon and education, 4) control	455 Adults	4 months	Low-income black women and white women receiving WIC in Genesee County, Michigan, 2011	None reported	To increase FV attitudes and intake through a coupon intervention and education combined
Not named (Los Angeles economic subsidy) (38,39)	Quasi-experimental, 3 arms: 1) WIC site no. 1 received supermarket voucher, 2) WIC site no. 2 received farmers market voucher, 3) WIC site no. 3 was control	454 Adults	6 months	Adult Hispanic women receiving WIC in Los Angeles, California, 2001	None reported	To increase FV intake through an economic subsidy for FV for postpartum WIC participants
Shop N Save (40)	Quasi-experimental, time series, no comparison	336 Adults	2011 and 2012 farmers market season (40 total market days)	Low-income, predominantly black women in rural South Carolina, 2011–2012	CBPR	To increase access to FV, increase use of food assistance, and improve revenue trends at a farmers market through a pricing intervention
**Redeemable coupons or vouchers for healthy foods and beverages targeting nonparticipants in food assistance programs**						
Not named (French supermarkets) (41,42)	RCT, 3 arms: 1) letter with social norm feedback, 2) letter plus financial incentive, 3) control	2,672 Adults	12 months	Low-income health-deprived adults in France, 2007–2009	None reported	To evaluate the impact of nutritional counseling alone, or counseling plus vouchers, on FV consumption and biomarkers
Not named (New York City farmers markets) (43)	Case-control, nonrandomized trial, 2 arms: 1) rebate, 2) control	169,485 Households	12 weeks	Low-income overweight Latino women with type 2 diabetes in New York City, 2011	None reported	To improve intake and purchasing of FV by a combined education and voucher intervention
Spend Study (44)	RCT, 2 arms: 1) rebate, 2) control	5,076 SNAP participants	4 weeks	Low-income, food-insecure households with 1 child or more aged ≤18 y in New Zealand, 2009–2010	None reported	To examine the effect of additional money (supermarket vouchers) on food expenditures in food-insecure households with children.
Trying Alternative Cafeteria Options in Schools (TACOS) (45–50)	RCT, 2 arms: 1) intervention; 2) control	54 male and female students	2 years	Secondary school students in Minneapolis–St Paul, Minnesota, 2000–2002	SCT	To increase availability and sales of low-fat food options in high school cafeterias
Not named (United Kingdom fruit juice delivery) (51)	RCT, 2 arms: 1) rewards intervention, 2) delayed intervention control	58 Adults	30 weeks	Low-income pregnant women in the United Kingdom	None reported	To increase fruit and fruit juice intake by pregnant women by using vouchers or counseling
What to Eat for Lunch study (52)	RCT, 2 arms: 1) intervention (2 phases: full intervention including voucher; partial intervention no voucher), 2) delayed treatment control	28 Adults	8 weeks	Overweight/obese hospital employees, majority black women, in Philadelphia, Pennsylvania, 2012	IMB	To promote healthy lunch purchases at work through combined mindful eating, initial price reductions, and online pre-ordering
**Cash Rebate**						
Not named (Boston social norm and rebate study) (53)	RCT, 3 arms: 1) letter with social norm feedback, 2) letter plus financial incentive, 3) control	2,672 Adults	6 months	White high-income hospital employees, Boston, Massachusetts, 2012–2013	None reported	To increase healthy food purchases through social norm feedback with and without a financial incentive
Healthy Food program (54,55)	Case-control, nonrandomized trial, 2 arms: 1) rebate, 2) control	169,485 Households	5 months	Members of a South African health plan, South Africa, 2009–2012	None reported	To examine the effect of price reductions for healthy food items on food purchases (healthy and unhealthy)
Healthy Incentives Pilot (HIP) (56,57)	RCT, 2 arms: 1) rebate; 2) control	5,076 SNAP participants	12-monthsintervention staggered over 3 waves	SNAP participants in rural, urban, and suburban communities in Hampden County, Massachusetts, 2011–2012	ET	To test the effect of a rebate on FV purchases on change in purchasing of FV
Not named (Philadelphia financial incentives) (58)	RCT, 2 arms: 1) treatment, 2) control	54 Men and women	3 months	Low-income, predominantly black, middle-aged adults in Philadelphia, Pennsylvania(year not provided)	None reported	To test the effects of financial incentives for the purchase of healthy foods and beverages on purchasing of healthy food items, dietary intake, household food environment, BMI
Rewards study (59–61)	RCT, 2 arms: 1) rewards intervention, 2) delayed intervention control	58 Adults	12-week pilot study of 26-week study duration in 4 phases	Low-income black adults in Philadelphia, Pennsylvania, 2010–2011	SEM	To assess impact of rewards-based incentives on purchases of fresh and frozen FV
**Disincentives for unhealthy food and beverage purchases, with and without incentives for healthy food and beverage purchases**						
Berkeley, California, excise tax on soda (62,63)	Natural experiment, pre–post with a comparison group (San Francisco, Oakland)	2,989 (different samples: pretax, 1,048; posttax, 1,941)	1 year	Low-income black and Hispanic population in Berkeley, 2014–2015	None reported	To evaluate the impact of the excise tax ($0.01/oz) on SSB prices and consumption
Danish saturated fat tax (64–67)	Natural experiment, pre–post assessments	2,577 Households, 1,293 total retailers	2 years	Danish consumers, 2010–2012	None reported	To estimate the impact of a saturated fat tax on consumption of saturated fat and other nontargeted dietary measures
Excise tax on SSBs in Mexico (68–71)	Natural experiment, pre–post assessments	>50,000 Households in Mexico, 14,784 with children	2 years	Mexican households, especially households with a child aged 2–5 y, 2012–2014	None reported	To determine the effect of the 1 peso/L tax on SSBs on SSB purchases
Not named (French food baskets) (72)	Experimental, controlled, 2 conditions tested: 1) FV subsidy only, 2) FV and other healthy food subsidy plus increased price on unhealthy products	128 Women	1 day	Low- and medium-income women, main food shoppers, in Grenoble and Lyon, France, 2008	None reported	To explore the effect of food price policies (taxes, subsidies) on expenditures for and nutritional quality of the food baskets chosen by low-income and medium-income households
Not named (Minneapolis financial incentives) (73)	RCT, 4 arms: 1) FV incentive, 2) restrictions, 3) both, 4) control	297 Adults	12 weeks	New SNAP participants in Minneapolis, Minnesota, 2013–2015	None reported	To determine if an FV financial incentive alone, prohibition of purchasing unhealthy foods with food benefits alone, both in combination, or control improves diet quality
Not named (Brussels University cafeteria study) (74)	Mixed-methods study with 2 phases (phase 1, French fries followed by short interviews; phase 2, fruit intervention followed by short interviews)	230 Students; sales data recorded on 2,300–2,930 sales for phase 1 and 3,235–3,802 during phase 2. Qualitative research: 230 students	10 weeks in 2 phases	University students eating in on-campus cafeteria, Brussels, Belgium, 2015	None reported	To examine the effect of a pricing intervention (tax on French fries and 10%–20% price subsidy on fruit) on students’ purchasing behavior

Abbreviations: BE, behavioral economics; BMI, body mass index; CBPR, community-based participatory research; ET, economic theory; FV, fruits and vegetables; IMB, information–motivation–behavioral skills theory; K–12, kindergarten through 12th grade; RCT, randomized controlled trial; SCT, social cognitive theory; SEM, social ecological model; SES, socioeconomic status; SM, social marketing; SNAP, Supplemental Nutrition Assistance Program; SSB, sugar-sweetened beverage; WIC, Special Supplemental Nutrition Program for Women, Infants, and Children.

At the consumer level, sample sizes ranged from 28 individual participants (What to Eat for Lunch study) to more than 50,000 households (Mexico excise tax study), with a median of 454 individuals sampled. Among the randomized controlled trials, the median sample size per study arm was 100 participants. The sample size or unit of randomization in some studies was based on clusters (eg, food stores) and not necessarily on individuals.

Almost all studies (n = 25) examined the impact of a pricing intervention alone or in combination with other strategies related to the stocking, sales, purchasing, or consumption of healthy foods. Most studies targeted low-income, disadvantaged populations (n = 18). Many studies (n = 12) targeted a specific population that was reached through the venue of the intervention (eg, a worksite, sports gym, school, swimming pool, hospital). 

### Interventions and strategies studied

Nearly all studies (n = 27) examined interventions that promoted healthy foods (Table 2). The most common types of foods promoted were fruits and vegetables (n = 20), particularly fresh produce, followed by low-sugar beverages (n = 10), and healthily prepared entrees and side dishes (n = 8). Only a few studies (n = 6) discouraged unhealthy foods, such as SSBs and foods high in saturated fat and sugar, as part of the intervention, generally by raising the prices of these foods.

**Table 2:** Intervention and Evaluation Strategies of Food and Beverage Pricing Studies, 2000–2016

Type of Study/Study Name	Type of Food or Beverage	Source	Intervention	Change of Availability, Location, or Labeling	Policy	Other	Impact Measure: Retail	Impact Measure: Consumer
**Financial discounts on healthy foods and beverages**								
Baltimore Healthy Carryouts (10–17)	Healthy prepared foods (side dishes, entrees) and beverages (reduced or no sugar)	8 Carryout restaurants	Reduced-price healthy combination meal	Increased stocking of healthy foods and beverages; revised menu board to highlight healthy foods and beverages (labeling, photos)	None	Nutrition education	Sales, revenues (carryout)	Psychosocial: none; behavioral: food purchasing; health: none
B’More Healthy Retail Rewards (18,19)	FV, low-sugar beverages, nutrient-dense foods, low-fat snacks, whole- grain products	24 Corner stores, 1 wholesaler	10%–30% Price discount on healthy food items at point of purchase from wholesaler	Increased stocking of promoted healthy foods; shelf labels and shelf talkers identifying healthy foods in store and at wholesaler	None	Nutrition education, media, and structural changes	Sales (retailer and wholesaler) and owner’s psychosocial factors	Psychosocial: food security, knowledge, self-efficacy, intentions; behavioral: food purchasing, dietary intake; health: BMI, food security
Not named (healthy foods at swimming pools) (20)	Healthy items meeting definition of “choose most often” (Alberta Nutritional Guidelines for Children)	2 Concession stands in an outdoor swimming pool facility	30% Discount on healthy foods	Heathy menu items emphasized with photographs, appealing names; signage large and close to cashier; price reduction for healthy foods indicated in signage	None	Staff training, taste testing of healthy foods	Sales, revenues, and gross profits	Psychosocial: none; behavioral: purchasing; health: none
HealthWorks (21–24)	Low-calorie fresh and prepared foods	6 Worksite cafeterias, vending machines	15% Price reduction on calorie-smart foods	Increased availability of healthy foods by at least 50% of all cafeteria and vending machine offerings; smaller portion sizes as substitutes; labeled calorie-smart items at point of purchase	None	Media, weight and activity self-monitoring, and nutrition education	None	Psychosocial: none; behavioral: stair use, absenteeism; health: BMI (measured height and weight)
Not named (Mississippi Healthy Beverages) (25)	Healthy beverages (water, 100% fruit juice, sports drinks)	18 School vending machines and stores	10%–25% Discount on healthy drinks	At least 50% of beverages sold in school should be water, 100% fruit juices, and sports drinks; passive marketing of beverages through changed facings and display cases	None	None	None	None
Not named (multi-component intervention in sports clubs) (26)	Fresh FV (fruit, salads, or salad sandwiches), non-SSBs	85 Sporting club canteens	Combination meals with FV products and water packaged together at a reduced price	Substituted higher fat and energy products with lower fat and energy products and introduced products lower in energy, fat, or sodium; positioned promoted foods at eye level, upper half of refrigerator, or on the counter; placed signage and posters to draw attention to FV products and non-SSBs	Written food and nutrition policy	Media, nutrition education, and training	Financial records of canteen revenues	Psychosocial: none; behavioral: purchasing; health: none
Supermarket Healthy Eating for Life (SHELf) trial (27–30)	FV (fresh, frozen, canned, and dried), low-calorie soft drinks, and water	2 Grocery stores, 1 in low-SES area, 1 in high-SES area	Reduce purchase of SSBs; 20% price discounts on all FV, low-calorie soft drinks, and water	Not done	None	Nutrition education	Sales data (on healthy fats, FV, healthy meats, and milk)	Psychosocial: none; behavioral: electronic sales data measured as a proxy for purchasing; health: none
Supermarket Healthy Options Project (SHOP) (31–34)	Healthy core foods and beverages that met Tick program criteria (total fat, saturated fat, *trans* fatty acids, sodium, added sugar, fiber, and calcium)	8 New Zealand supermarkets, all part of the same chain	12.5% Discount for healthy foods that met Tick program criteria	Not done	None	Nutrition education	Sales data (cereals, healthy fats, FV, healthy meats, and milk)	None
Not named (Lima University cafeteria study) (35)	Fresh fruits	1 University cafeteria	33% Reduction in fruit price	Relocated fruit items closer to cash register; nutrition benefit sign at fruit container; poster promoting the item, and price tags	None	None	Cafeteria sales data (unit, ratio of fruit purchased)	Psychosocial: reasons for not purchasing fruits; behavioral: none; health: none
**Redeemable coupons/vouchers for healthy food and beverage items targeting recipients of food assistant programs**								
Farmers Market Fresh Fund Incentive Program (36)	Fresh FV, healthy packaged foods (eggs, bread, and meat)	5 Farmers markets	Fresh Fund incentive match tokens (up to $20/month) for SNAP recipients	Not done	None	Media campaign	Revenue of Fresh Fund and non–Fresh Fund purchases	Psychosocial: perceived healthfulness of diet; behavioral: frequency of use of farmers market, money spent on FV per week, daily servings of FV consumed; health: none
Project FRESH (Farm Resources Encouraging and Supporting Health) (37)	Fresh FV	City farmers markets (no specific number)	$20 Coupons for farmers markets	Not done	None	Nutrition education	None	Psychosocial, attitudes toward FV; behavioral, intake of FV (daily, perceived change); health, none
Not named (Los Angeles economic subsidy) (38,39)	Fresh FV	City supermarkets, farmers markets	$10/Week redeemable voucher	Not done	None	None	Sales of beverages (monthly)	None
Shop N Save (40)	Fresh FV	1 Farmers market	$5 Matching coupons for participants of SNAP, WIC, Senior or WIC FMNP	Not done	None	None	Consumer sales receipts	None
**Redeemable coupons or vouchers for healthy food and beverage items targeting nonparticipants in food assistance programs**								
Not named (French supermarkets) (41,42)	Fresh FV	22 Supermarkets	Supermarket vouchers, (10 Euros/person in the household for fresh FV)	Not done	None	Nutrition education	Availability of calorie-smart foods	Psychosocial: none; behavioral: self-reported voucher use, FV consumption; health: BMI, blood pressure, blood measures of vitamin C, serum β carotene
Not named (New York City farmers markets) (43)	Fresh FV	1 Farmers market	$6 Vouchers for farmers market purchases	Not done	None	Nutrition education	None	Psychosocial: none; behavioral: frequency of use of farmers market, intake of FV; health: BMI, LDL cholesterol, HbA1c, and BP measures
Spend Study (44)	FV, healthy grocery food items, and highly processed foods	Supermarket (no specific number), identified as most frequently used	$5 Supermarket voucher per individual in the household (average $17/household/ wk; voucher was not specific for any food or food group)	Not done	None	Main food preparer received reminder text messages, emails, or telephone calls	None	Psychosocial: none; behavioral: food group purchases, total household food expenditure; health: none
Trying Alternative Cafeteria Options in Schools (TACOS) (45–50)	Low-fat foods (≤5 g/serving)	20 High school cafeterias	Coupon (one-time free low-fat food), raffle for each low-fat food purchase	Increased availability of low-fat foods by 30% relative to baseline	None	Nutrition education, media campaigns	Unit sales, total revenue from low-fat foods sales	Psychosocial: perceived environment, behavioral intentions; behavioral: self-reported food choices; health: none
Not named (United Kingdom fruit juice delivery) (51)	Fruit, 100% fruit juice	Delivery system	Vouchers exchanged for 100% fruit juice by the local milk delivery service; received 21 Euros/week for 30 weeks	Not done	None	Nutrition education	None	Psychosocial: barriers to eating fruit; behavioral: dietary intake (frequency of fruit consumption in past 7 days); health: serum β carotene
What to Eat for Lunch study (52)	Low-calorie/low-fat cafeteria lunch	1 Hospital cafeteria	Vouchers to use on lunch purchases (20 $1.25 vouchers for 4 wk)	Online pre-ordering system that provided calorie and fat content; default option was lower calories/fat	None	Nutrition education	Food purchases; fat and calorie content of each item	Psychosocial: mindful eating questionnaire; behavioral: none; health: BMI, blood measures (HbA1c, triglycerides, HDL cholesterol, LDL cholesterol)
**Cash rebate**								
Not named (Boston social norm and rebate study) (53)	Healthy foods on menu (FV, whole grains, lean protein, low-fat dairy); avoiding saturated fat and calories	1 Hospital cafeteria	Monthly financial award ($5–$30) based on proportion of green-labeled products purchased	Traffic-light system menu labeling	None	Nutrition education	Sales data from cafeteria cash registers	Psychosocial: none; behavioral: purchases of green-labeled foods; health: none
Healthy Food program (54,55)	FV, other healthy foods, less-desirable foods and beverages, and neutral foods	432 Supermarkets	10%–25% Rebate for healthy food purchases	In-store labeling identifying eligible foods, which were also marked on store receipts	None	None	Scanner data from credit card charges at the supermarket	Psychosocial: none; behavioral: consumption of FV, whole grains, salt-added foods, salty and sweet snacks; health: BMI measure
Healthy Incentives Pilot (HIP) (56,57)	Fresh, canned, frozen, and dried FV	All SNAP-authorized retailers in Hampden County, including supermarkets, superstores, grocery and food specialty stores, convenience stores, and farmers markets	30% Rebate on total FV purchased using EBT cards for 12-month period	Not done	None	Nutrition education	FV sales	Psychosocial: attitudes and perceptions about FV; behavioral: diet (24-hour recall, FV screener); health: none
Not named (Philadelphia financial incentives) (58)	FV (fresh, canned, frozen), low-calorie beverages, and low-energy–dense foods	All food stores providing receipts	Financial incentive ($1) for every healthy food item purchased over 3 months, $100 maximum	Not done	None	Nutrition education	None	Psychosocial: attitudes toward grocery stores (baseline only); behavioral: dietary intake (3-day food record), home food environment; health: BMI, waist circumference measures
Rewards study (59–61)	Fresh or frozen FV (defined by WIC guidelines)	1 Large supermarket in a predominantly black census tract	50% Rebate on dollar amount spent on fresh or frozen FV, reduced to 25% during a tapering phase	Not done	None	Communication materials, nutrition education	Point-of-sale data by family provided by supermarket	Psychosocial: perceptions of behaviors resulting from study (post); behavioral: none; health: none
**Disincentives for unhealthy food and beverage purchases, with and without incentives for healthy food and beverage purchases**								
Berkeley, California, excise tax on soda (62,63)	SSBs	All beverage retailers in Berkeley, California	Not done	Not done	SSB $0.01-per-ounce tax on distributors	None	Change in price pre- and post-tax	Psychosocial: awareness of tax; behavioral: change in consumption of SSBs and water; health: none
Danish saturated fat tax (64–67)	Foods containing saturated fat (eg, butter, blends, margarine, oil, meat, sour cream)	1,293 Food retailers in Denmark	Not done	Not done	25% Tax on foods high in saturated fats	None	Monthly sales and revenues	Psychosocial: none; behavioral: purchase of food, food intake; health: modeled estimation of mortality from NCDs, BMI measured
Excise tax on SSBs in Mexico (68–71)	SSBs, nonessential high energy, high density foods	16,000 Food retailers	Not done	Not done	1 Peso per liter tax on SSBs	None	Change in prices of SSBs	Psychosocial: none; behavioral; purchases of SSBs and other nonessential foods; health: none
Not named (French food baskets) (72)	43 Foods were classified as FV, 24 as other healthy products, 51 as neutral, 62 as unhealthy	Real-life laboratory, online order (foods received)	Subsidy for FV (30% discount), tax on unhealthy products (30% increase)	Price change identified on screen (old price crossed out)	Assessed impact of subsidies and taxes on food basket selections	None	None	Psychosocial: none; behavioral: purchasing of food baskets; health: none
Not named (Minneapolis financial incentives) (73)	FV, SSBs, sweet baked goods, candies	All SNAP retailers	30% financial incentive for FV purchased using food benefits; restriction (not allowed to buy SSBs, sweet baked goods, or candies) with food benefits	Not done	None	Training	None	Psychosocial: food security; behavioral: food intake and diet quality; health: none
Not named (Brussels University cafeteria study) (74)	French fries (unhealthy food product) and fruit (healthy food product)	1 On-campus, Brussels University restaurant	Total meal price increases of 10% and 20% when choosing French fries, and 10% and 20% meal price decreases when choosing fruit for dessert	Point-of-purchase posters and information boards	None	Social media	French fries and fruit sales counts relative to total number of items sold	Psychosocial: food price, food preference, food knowledge, body satisfaction, perception of availability and access of food; behavioral: none; health: none

Abbreviations: BP, blood pressure; BMI, body mass index; EBT, electronic benefit transfer; FMNP, Farmers Market Nutrition Program; FV, fruits and vegetables; HbA1c, hemoglobin A1c; HDL, high-density lipoprotein; LDL, low-density lipoprotein; NCD, noncommunicable disease; SES, socioeconomic status; SNAP, Supplemental Nutrition Assistance Program; SSB, sugar-sweetened beverage; WIC, Special Supplemental Nutrition Program for Women, Infants, and Children.

The types of food sources targeted varied and included grocery stores and supermarkets (n = 7), all retailers in a setting (eg, city, neighborhood) (n = 6), farmers markets (n = 5), worksite cafeterias and school cafeterias (n = 5), food delivery services (n = 2), carryout restaurants (n = 1), corner stores (n = 1), and other types of retailers. The number of the food sources intervened in for each study also varied, from one to many thousands, because some studies implemented the strategy city-wide (median, 5 food source locations). Pricing interventions also differed between studies. Nine studies emphasized price discounts on healthy foods and beverages, ranging from 10% to 33%. Four studies provided coupons or vouchers of $5 to $20 to food assistance recipients. Six studies provided coupons or vouchers of $1 to $22 to the general population. In 5 studies, the pricing intervention was a cash rebate. The amount of the rebate took many different forms, such as a straight percentage off or a price reduction up to a certain limit. Six studies tested price increases on unhealthy foods, half of which included a price reduction on healthier foods. Three of these studies were of local or federal taxes, including taxes on SSBs.

Six of the 30 studies sought to change the availability of healthy or unhealthy foods. Only 2 of the studies changed the physical location of foods as a means of increasing their uptake by consumers. Eleven of the 30 studies implemented labeling to identify healthy versus unhealthy food choices. Most labeling approaches occurred in studies (n = 8) centered on the promotion of healthy foods and beverages. Five of the 30 studies used a policy approach, and 4 studies involved taxes at the city or national level.

### Evaluation strategies

Most studies (n = 17) reported no formative research (Appendix Table 2). When formative research was conducted, it consisted of qualitative information gathering (n = 3), structured survey data collection (n = 4), or a pilot study (n = 6).

Most studies (n = 20) reported conducting a feasibility assessment, which consists of assessment of economic or cultural acceptability, operability, or perceived sustainability. However, feasibility assessment varied greatly among studies in terms of rigor and scope.

Process evaluation assesses how well an intervention was implemented according to the study plan and is usually assessed in terms of reach, dose delivered, and fidelity (75). Most studies (n = 18) reported no process evaluation. Two studies reported conducting extensive process evaluations that assessed reach, dose delivered, and fidelity (10,19).

Most studies (n = 20) assessed the impact of the intervention at the retail level (Table 2). Of these 20 studies, 15 collected data on sales of specific promoted foods. Other studies looked at changes in revenues, food availability, purchasing data, and changes in prices, although these measures were used in only 2 studies (36,40).

We examined impact assessment at the consumer level in 3 different domains: psychosocial, behavioral, and health outcomes. More than half (n = 17) of the studies reviewed included no consumer-level psychosocial assessment. Of those that did, measures used varied and included knowledge, self-efficacy, intentions for healthier behaviors, perceived healthfulness of the diet and affordability of healthy foods, perception of barriers to eating healthy, and food security.

Most studies (n = 24) described consumer-level behavioral assessment, most often measurements of food purchasing and consumption. Only 10 of the 30 studies reviewed measured any type of consumer-level health outcome. Most commonly, change in body mass index (BMI [kg/m^2^]) was assessed (7 studies), followed by blood work (4 studies).

### Study results reported and study implications

We found little consistency in study results reported for feasibility and process measures (Appendix Table 2). Where reported, feasibility of pricing interventions was moderate to high. Pricing interventions were acceptable and generally were implemented as planned. In 1 study (21), the pricing intervention was not implemented at any site because of food managers’ concerns about profit loss. Where extensive process results were reported (2 studies), implementation quality was generally described as moderate (10,19).

Of the studies (n = 21) that measured an intervention’s impact at the retail level, the most common effects reported were increased sales of healthy foods (7 studies) (11,19,20,35,45,58,59, improved revenues or total profits (4 studies) (11,25,36,40), increased stocking of healthier foods (4 studies) (19,21,26,74, decreased sales of unhealthy foods (3 studies) (25,64,68), and increased sales of healthy foods as a ratio to unhealthy foods (2 studies) (10,55) (Table 3). All 16 studies that reported effects at the retail level found a positive impact on either stocking or sales. In summary, sales of units of healthy foods and beverages increased from 15% (19) to 1,000% (25), and sales of unhealthy foods and beverages decreased from 5% (64) to 47% (69). Stocking of healthy foods increased from 40% (19) to 63% (26) in response to pricing interventions (Table 3).

**Table 3:** Findings from Pricing Intervention Studies, 2000–2016

Type of Study/Study Name	Retail Stocking and Sales	Consumer Psychosocial	Consumer Behavioral	Consumer Health Outcomes	Sustainability^a^	Quality of Research^b^
**Studies of financial discounts on healthy foods and beverages**						
Baltimore Healthy Carryouts (10–17)	296% Increase in units of healthy sides and beverages sold; total revenues (healthy foods only: 39%, healthy foods and beverages: 173%) among intervention carryouts from baseline	—c	450% Increase in purchasing of promoted foods	—c	Moderate	9
B’More Healthy Retail Rewards (18,19)	40%–61% Increase in stocking score in all intervention groups; 15% increase in sales of snack foods for combined intervention group	—c	—c	—c	—d	8
Not named (Healthy Foods at Swimming Pools) (20)	30% Increase in sales of healthy items during discounted period in a subsample	—c	Overweight or obese patrons and males were more sensitive to signage plus taste testing plus pricing intervention	—c	—d	6
HealthWorks (21–24)	50% Increase in availability healthy foods; no impact on pricing (not implemented)	—c	Increase in frequency of self-weighing	No difference on weight outcomes over the 2-year period	Moderate; all components but pricing intervention were sustained.	9
Not named (Mississippi Healthy Beverages) (25)	Increase in sales and profits of 125% on units of water, 134% on units of sports drinks, >1,000% increase on units of fruit juice in most schools; 55% decrease on unit sales of soda in 9 schools	—c	—c	—c	—d	5
Not named (multi-component intervention in sports clubs) (26)	63% Increase in availability of FV	—c	60% Increase in club members purchasing FV; 13.4% increase in club members purchasing non-SSBs	—c	—d	7
Supermarket Healthy Eating for Life (SHELf) trial (27–30)	—c	Increased perceptions for healthy eating, cooking, and eating healthy in all interventions; no differences among intervention groups	35% Increase in purchasing of FV and 15% increase in non-SSB purchases in price reduction alone and price plus behavior arms; impact not maintained at 6-months postintervention; no effect on water or low-calorie beverages	—c	Low	10
Supermarket Healthy Options Project (SHOP) (31–34)	—c	—c	10% Increase in purchases of healthy items (0.79 kg/wk) in discount group at 6 months; no effect reported on education-only group at 6 months	—c	High; after cessation of pricing discount, discount group maintained FV and other healthy food purchasing at 12 months	8
Not named (Lima University cafeteria study) (35)	135% Increase in units of fruit sales	Most common reason to not purchase fruit was preference for unhealthy snack foods	57% Increase in purchasing of fruits among females and tripled among males	—c	—d	2
**Redeemable coupons or vouchers for healthy foods and beverages targeting participants in food assistance programs**						
Farmers Market Fresh Fund Incentive Program (36)	74% Increase in revenue of farmers market vendors	Increase in perception of healthfulness and likelihood to continue shopping at a farmers market	24% Increase in people consuming 5 or more FV servings per day	—c	High; participating farmers markets continued to offer incentives to consumers	6
Project FRESH (Farm Resources Encouraging and Supporting Health) (37)	—c	Increase in attitudes and beliefs regarding FV in intervention group	140%–640% Increase in FV intake score in intervention groups	—c	—d	7
Not named (Los Angeles economic subsidy) (38,39)	—c	—c	30% Increase in FV intake in intervention groups	—c	High; increase in FV intake sustained 6 months after intervention	7
Shop N Save (40)	43% Increase in food assistance revenues at farmers markets	—c	—c	—c	High; Double Buck program to be implemented by South Carolina	5
**Redeemable coupons or vouchers for healthy foods and beverages targeting nonparticipants in food assistance programs**						
Not named (French supermarkets) (41,42)	—c	—c	33% Increase in FV intake in voucher group and 32% for advice group	No change in serum vitamin C and β carotene levels; no difference in other health measures by group	—d	6
Not named (New York City farmers markets) (43)	—c	Decrease in reported difficulty of affording FV	20% Increase in FV intake (servings/d)	Decreased BMI and HbA1c, but no significant difference by group	—d	7
Spend Study (44)	—c	—c	No difference in expenditures on other food groups (ie, FV, meat and poultry, dairy)	—c	—d	7
Trying Alternative Cafeteria Options in Schools (TACOS) (45–50)	Increase in sales of low-fat foods (at year 1, 27.5%; at year 2, 33.6%); no significant change in total revenues	Increased perception of more low-fat food availability; no change in environmental or behavioral intentions	No impact on food choices	—c	—d	10
Not named (United Kingdom fruit juice delivery) (51)	—c	Taste and appetite were barriers to eating fruit	59.1% Increase in fruit juice intake (net percentage of consumption)	Increase in serum βcarotene	—d	8
What to Eat for Lunch study (52)	—c	Increased scores for mindful eating behaviors	8% Decrease in total calories and 6% decrease in total fat in food purchases using pre-ordering only (no vouchers) compared with baseline and discount phase	Decreased weight, HbA1c, and lipid profiles from pre to post, but not significant	High; in partial intervention phase without any financial incentives, participants still bought foods with lower calorie and fat content	9
**Cash rebate**						
Not named (Boston social norm and rebate study) (53)	—c	—c	2.2% Increase in purchasing of green-label foods; no difference between intervention arms; increase in healthy food choices in social norms and small financial incentives	—c	Low; intervention effect did not persist 3 months after completion of trial	10
Healthy Food program (54,55)	—c	—c	9.3% Increase in healthy food to total food expenditures; 63% increase in consumption of FV and 195% of whole-grain foods; 68% decrease in consumption of unhealthy foods (high-sugar or high-salt foods, fried foods, processed meats, fast food)	No effect on obesity	—d	7
Healthy Incentives Pilot (HIP) (56,57)	Increase in FV sales in large grocery store (qualitative finding)	Increase in attitude toward FV over time	40% Increase in FV intake; 10% decreased intake of refined grain; increase in HEI–2010 score	—c	—d	9
Not named (Philadelphia, financial incentives) (58)	—c	—c	10% Increase in protein intake, 28% in calcium intake, and 60% in daily vegetable intake; increase in household food environment	Slight decrease in BMI in both groups	—d	6
Rewards study (59–61)	—c	Increased perception of buying more FV	25%–30% Increase in purchasing of FV servings/wk (30% increase in vegetable and 25% increase in fruit servings/wk); effect not sustained when incentive was reduced	—c	Low; changes not maintained during tapering period	7
**Disincentives for unhealthy food and beverage purchases, with and without incentives for healthy food and beverage purchases**						
Berkeley, California, excise tax on soda (62,63)	9% Increase in SSB retail prices in Berkeley	Increased knowledge about the tax	21% increased consumption of SSBs; 63% increased water consumption	—c	High; tax is still in effect and Berkeley City Council allocated $1.5 million to fund program to reduce SSB consumption	6
Danish saturated fat tax (64–67)	5% Decrease in sales of ground beef and creams	—c	4% Increase in saturated fat intake; 1% increase in salt intake; 9% increase in FV intake	Increase in deaths from cardiovascular disease (modeled)	Low; tax is no longer in effect	5
Excise tax on SSBs in Mexico (68–71)	47% Decrease in sales of taxed foods; no change in sales of untaxed foods	—c	12% Decrease in SSB purchases; 5% decrease in nonessential food purchases	—c	High; tax is still in effect	5
Not named (French food baskets) (72)	—c	—c	25%–30% Increase in quantity of FV purchased among low- and middle-income shoppers; 52% decrease in unhealthy food expenditures among middle-income shoppers for nutrient profile condition	—c	—d	4
Not named (Minneapolis financial incentives) (73)	—c	Increased food security in all groups	2%–6% Decreased energy intake (incentive, 2%; restriction, 6%; combined, 6%); 66% increased intake of fruit; 8% increase in HEI–2010 score	—c	—d	9
Not named (Brussels University cafeteria study) (74)	Increase in availability of healthy foods (qualitative result: perception of students that influenced their food choices)		10.9%–21.8% decrease in French fries purchases; 25.1%–42.4% increase FV purchases	—c	Moderate; students believed that fruit price reduction could be sustained in the long term	3

Abbreviations: —, not assessed; BMI, body mass index; FV, fruits and vegetables; HbA1c, hemoglobin A1c; HEI, Healthy Eating Index; SNAP Supplemental Nutrition Assistance Program; SSB, sugar sweetened beverage.

a Sustainability was scored as low (no mention of continuing the pricing interventions after the study has ended), moderate (few components of pricing interventions remained after the study has ended), or high (most components of pricing interventions remained after the study has ended).

b Ranked on a scale of 0 to 10.

c No assessment of interests was mentioned.

d Not reported.

Only 13 studies reported any assessment of the impact of interventions on consumer psychosocial factors. Four studies found improved perceptions related to healthy eating (27,37,56,74). Three studies indicated that consumers improved their perception of healthfulness or availability of fruits and vegetables (36,45,60). Two studies found that consumers were more likely to shop at farmers markets (36,43).

Most (n = 23) studies assessed the impact of interventions on consumer behavior. Thirteen studies found increases in the consumption of healthy foods and beverages associated with the intervention (36–38,40,41,43,51,54,56,58,62,65,73), and 8 studies found increases in purchasing of healthy foods (10,26,27,31,35,53,54,59). Four studies found a reduction in the purchasing of unhealthy foods (52, 67, 71, 73). Four studies found a reduction in the consumption of unhealthy foods (55, 56, 64, 72). Two studies reported no effect on healthy food purchasing (44,45), and 1 found no impact on healthy beverage consumption (27). Overall, the pricing interventions, alone or in combination with other approaches, appeared to be successful in changing consumer behavior.

Although few studies (n = 8) assessed health-related outcomes at the consumer level, 5 found no impact on weight (20,22,26,41,58); 2 found no impact on various serum vitamin measures when comparing control and intervention groups over time (41,51).

Of the 14 studies that reported on sustainability of the intervention, 10 stated moderate to high sustainability through statewide or citywide implementation of the intervention (11,40), food policies that are still in progress (62,68), and continued interest of the participants (21,31,36,38,52,74).

The mean score for quality of research measures was 6.9 (standard deviation, 2.0), on a scale of 0 to 10 points (Table 3). Randomized controlled trials received higher scores than studies without a comparison group. Common study limitations included short intervention duration, possible biases in self-reporting, use of nonvalidated assessment tools, and lack of power and external validity of the findings.

## Discussion

To our knowledge, this is the first systematic review to evaluate the effectiveness of pricing incentives and disincentive strategies on availability, purchasing, and consumption of healthy and unhealthy foods and beverages in various settings, including field intervention studies and natural experiments. The various pricing intervention strategies that sought to improve access to healthy food and beverage choices were successful. This result has been reported by other systematic reviews where subsidies on fruits and vegetables increased the purchasing and consumption of healthy foods (2,76,77). However, only one study evaluated the impact of fruit and vegetable subsidies from the perspective of retailers (74). Findings that the pricing interventions generally increased stocking and sales of promoted foods and beverages are encouraging. There is a need to consistently demonstrate these effects (particularly in terms of sales and revenues), to build support from food retailers and vendors. We recommend that additional studies be conducted to demonstrate beneficial effects of pricing interventions on sales, and especially on profits and total retail revenues.

Pricing intervention strategies appeared to positively affect consumer-level behavior, with most studies reporting increases in purchasing and consumption of healthy foods or beverages or decreased purchasing and consumption of unhealthy foods or beverages. We found no strong pattern to indicate that one type of pricing intervention worked better than another — all appeared to be effective. Additional studies and meta-analyses are needed to differentiate the potential impact of particular pricing interventions and policies over others. Only 2 studies changed the placement of foods in a store or market to make healthy choices more evident (26,35). This strategy can be effective, particularly when coupled with a pricing intervention and should be tested in future trials.

Most studies promoted fresh fruits and vegetables. However, these foods, especially for small retailers located in low-income settings, may be hard to source, have high perishability, and raise concerns about safety and handling (12,78). In addition, it is arguable that focusing on fresh fruits and vegetables alone is unlikely to make a substantial dent in diet-related chronic diseases (79). Pricing intervention trials should be broadened to include a range of healthy foods and beverages, including frozen, and even canned, foods.

Very few studies of food pricing interventions we reviewed discouraged unhealthy foods. Formative research has revealed that it is easier to convince food source owners to optimize the purchase of healthy foods than to get them to discourage the purchase of unhealthy foods (80,81). However, without some emphasis on decreasing consumer uptake of unhealthy foods and beverages, interventions that focus on healthy foods presume a substitution effect that may not exist. An exception to this concern are taxes on junk food and SSBs that have been adopted in recent years. Additional studies are needed in real community settings, testing both subsidies of healthy foods and beverages and increased prices of unhealthy foods and beverages.

Labeling foods that are part of pricing interventions appears to be a low-cost and effective way to draw attention to these foods. However, few studies reported labeling unhealthy foods. We need additional experimental trials in community food source settings that involve labeling both healthy and unhealthy foods and beverages.

Many of the studies we reviewed were small (ie, involved fewer than 50 respondents per treatment group), which raises concerns regarding enough statistical power to detect the true effect of the intervention. Future studies should be powered to find statistical differences between evaluation groups at the food source and consumer levels. Researchers can improve the transferability of their findings by disclosing how the sample size was determined.

Few studies included in the review attempted to assess the impact of pricing interventions on health outcomes. It may be unrealistic to hope to see the impact of policy and environmental interventions of this nature on health outcomes. However, natural experiments, given sufficient study duration, may be able to assess the impact of some of the large city-based policy initiatives, such as soda taxes. The average intervention duration was less than 1 year, and most lasted only a few months. Pricing intervention studies of longer duration are needed to track effects on health outcomes, and not just at the behavioral level.

The lack of formative research for most trials is of concern, especially in those studies that targeted specific populations. Even when formative research was conducted, it was minimal and not reported in any detail. Future pricing interventions should be based on solid formative research, and these findings should be reported in the published literature. Process evaluation of any form was rarely conducted in these studies. This is a major limitation of these studies, as it is of any intervention that neglects to collect process data (75). Failure to include process evaluation means that whether the failure of the intervention was because it was inherently flawed or because the intervention was not implemented as intended cannot be understood. Process evaluation data should be collected to assess implementation for all future pricing interventions. Several studies emphasized the importance of assessing the substitution effect (using savings from discounts to purchase other less healthy foods) and the compensation effect (purchasing more healthy foods but not reducing total energy intake) of pricing interventions on food purchases and dietary intakes (28,63,70). However, such assessment was not done in any of the studies reviewed and remains a major gap in this literature. Finally, a major gap in the studies reviewed is any type of uniform attention to consumer psychosocial outcomes. We recommend developing a core set of psychosocial measures for these types of intervention trials and recommend that they be based on theoretical frameworks.

This systematic review has several limitations. First, we focused exclusively on peer-reviewed literature. It is possible that additional, unpublished trials have been conducted. Second, some of the characteristics of specific trials that we marked as “not assessed” may have been assessed (eg, conducting formative research, process evaluation, cost-effectiveness) but were not published in peer-reviewed literature. This information may have been available in gray literature reports, on websites, or in other unexamined documents and thus were not included in this review. However, our use of only peer-reviewed literature helps to ensure a reasonable quality level of the research reported. Third, the use of only peer-reviewed literature may lead to publication bias, because studies with negative or null outcomes are less likely to be published. Fourth, our quality of study criteria did not include a measure of number of community venues for implementation sites. Nevertheless, we used these criteria to ensure comparability to previous studies (77). Finally, we did not conduct a meta-analysis to evaluate the pooled effectiveness of each pricing intervention strategy. Thus, the statement that one pricing strategy was no more effective than any other is based on the synthesis of the results and should be interpreted with caution. Because each pricing intervention strategy assessed different outcomes, it was challenging to compare the effect sizes of the studies and assess treatment effect. We included many studies outside the United States to enhance generalizability.

Pricing incentives and disincentive strategies to affect access, purchasing, and consumption of healthy and unhealthy foods and beverages in high-income and medium-income countries provide an evidence-based approach to improve healthy food access at the retail level and consumer purchasing and consumption (individual-level) behaviors. Most studies reviewed promoted fresh produce, although few discouraged purchasing and consumption of unhealthy foods. Further research that uses robust study designs and measurements are needed in real community settings to simultaneously test subsidies of healthy foods and beverages and the effects of increased costs of unhealthy foods and beverages.

## Appendix

Pricing Strategies to Encourage Availability, Purchase, and Consumption of Healthy Foods and Beverages: A Systematic Review

This appendix is available for download as a Microsoft Word document.

## References

[1] Powell LM , Chriqui JF , Khan T , Wada R , Chaloupka FJ . Assessing the potential effectiveness of food and beverage taxes and subsidies for improving public health: a systematic review of prices, demand and body weight outcomes. Obes Rev 2013;14(2):110–28. 10.1111/obr.12002 23174017 PMC3556391

[2] Thow AM , Downs S , Jan S . A systematic review of the effectiveness of food taxes and subsidies to improve diets: understanding the recent evidence. Nutr Rev 2014;72(9):551–65. 10.1111/nure.12123 25091552

[3] Andreyeva T , Long MW , Brownell KD . The impact of food prices on consumption: a systematic review of research on the price elasticity of demand for food. Am J Public Health 2010;100(2):216–22. 10.2105/AJPH.2008.151415 20019319 PMC2804646

[4] Powell LM , Chaloupka FJ . Food prices and obesity: evidence and policy implications for taxes and subsidies. Milbank Q 2009;87(1):229–57. 10.1111/j.1468-0009.2009.00554.x 19298422 PMC2879182

[5] Wilde PE , Llobrera J , Valpiani N . Household food expenditures and obesity risk. Curr Obes Rep 2012;1(3):123–33. 10.1007/s13679-012-0022-y

[6] Eyles H , Ni Mhurchu C , Nghiem N , Blakely T . Food pricing strategies, population diets, and non-communicable disease: a systematic review of simulation studies. PLoS Med 2012;9(12):e1001353. 10.1371/journal.pmed.1001353 23239943 PMC3519906

[7] Mytton OT , Clarke D , Rayner M . Taxing unhealthy food and drinks to improve health. BMJ 2012;344(may15 2):e2931. 10.1136/bmj.e2931 22589522

[8] Shemilt I , Marteau TM , Smith RD , Ogilvie D . Use and cumulation of evidence from modelling studies to inform policy on food taxes and subsidies: biting off more than we can chew? BMC Public Health 2015;15(1):297. 10.1186/s12889-015-1641-5 25881318 PMC4381483

[9] An R . Effectiveness of subsidies in promoting healthy food purchases and consumption: a review of field experiments. Public Health Nutr 2013;16(7):1215–28. 10.1017/S1368980012004715 23122423 PMC3898771

[10] Lee-Kwan SH , Goedkoop S , Yong R , Batorsky B , Hoffman V , Jeffries J , Development and implementation of the Baltimore healthy carry-outs feasibility trial: process evaluation results. BMC Public Health 2013;13(1):638. 10.1186/1471-2458-13-638 23837722 PMC3716976

[11] Lee-Kwan SH , Bleich SN , Kim H , Colantuoni E , Gittelsohn J . Environmental intervention in carryout restaurants increases sales of healthy menu items in a low-income urban setting. Am J Health Promot 2014;29(6)357–64. 10.4278/ajhp.130805-QUAN-408 24968184

[12] Noormohamed A , Lee SH , Batorsky B , Jackson A , Newman S , Gittelsohn J . Factors influencing ordering practices at Baltimore City carryouts: qualitative research to inform an obesity prevention intervention. Ecol Food Nutr 2012;51(6):481–91. 10.1080/03670244.2012.705732 23082919

[13] Lee-Kwan SH , Yong R , Bleich SN , Kwan NH , Park JH , Lawrence R , Carry-out restaurant intervention increases purchases of healthy food. J Hunger Environ Nutr 2015;10(4):456–66. 10.1080/19320248.2015.1045673

[14] Jeffries JK , Lee SH , Frick KD , Gittelsohn J . Preferences for healthy carryout meals in low-income neighborhoods of Baltimore city. Health Promot Pract 2013;14(2):293–300. 10.1177/1524839912465290 23182863

[15] Lee SH , Hoffman VA , Bleich SN , Gittelsohn J . Frequency of visiting and food dollars spent at carryouts among low-income, urban African American adults. J Hunger Environ Nutr 2012;7(4):459–67. 10.1080/19320248.2012.735220

[16] Lee SH , Rowan MT , Powell LM , Newman S , Klassen AC , Frick KD , Characteristics of prepared food sources in low-income neighborhoods of Baltimore City. Ecol Food Nutr 2010;49(6):409–30. 10.1080/03670244.2010.524102 21359162 PMC3043356

[17] Hoffman VA , Lee SH , Bleich SN , Goedkoop S , Gittelsohn J . Relationship between BMI and food purchases in low-income, urban African American adult carry-out customers. J Hunger Environ Nutr 2013;8(4):533–45. 10.1080/19320248.2013.816985

[18] Budd N , Cuccia A , Jeffries JK , Prasad D , Frick KD , Powell L , B’More Healthy: Retail Rewards — design of a multi-level communications and pricing intervention to improve the food environment in Baltimore City. BMC Public Health 2015;15(1):283. 10.1186/s12889-015-1616-6 25885923 PMC4379588

[19] Budd N , Jeffries JK , Jones-Smith JC , Kharmats AY , McDermott AY , Gittelsohn J . Store-directed price promotions and communications strategies improve healthier food supply and demand: impact results from a Baltimore City store-intervention trial. Public Health Nutr 2016. 28222818 10.1017/S1368980017000064 PMC5725746

[20] Olstad DL , Goonewardene LA , McCargar LJ , Raine KD . Choosing healthier foods in recreational sports settings: a mixed methods investigation of the impact of nudging and an economic incentive. Int J Behav Nutr Phys Act 2014;11(1):6. 10.1186/1479-5868-11-6 24450763 PMC3901328

[21] Linde JA , Nygaard KE , MacLehose RF , Mitchell NR , Harnack LJ , Cousins JM , HealthWorks: results of a multi-component group-randomized worksite environmental intervention trial for weight gain prevention. Int J Behav Nutr Phys Act 2012;9(1):14. 10.1186/1479-5868-9-14 22340088 PMC3305385

[22] VanWormer JJ , Linde JA , Harnack LJ , Stovitz SD , Jeffery RW . Weight change and workplace absenteeism in the HealthWorks study. Obes Facts 2012;5(5):745–52. 10.1159/000345119 23108493 PMC4032064

[23] VanWormer JJ , Linde JA , Harnack LJ , Stovitz SD , Jeffery RW . Self-weighing frequency is associated with weight gain prevention over 2 years among working adults. Int J Behav Med 2012;19(3):351–8. 10.1007/s12529-011-9178-1 21732212 PMC3474347

[24] VanWormer JJ , Linde JA , Harnack LJ , Stovitz SD , Jeffery RW . Is baseline physical activity a determinant of participation in worksite walking clubs? Data from the HealthWorks Trial. J Phys Act Health 2012;9(6):849–56. 10.1123/jpah.9.6.849 21952267 PMC3489008

[25] Brown DM , Tammineni SK . Managing sales of beverages in schools to preserve profits and improve children’s nutrition intake in 15 Mississippi schools. J Am Diet Assoc 2009;109(12):2036–42. 10.1016/j.jada.2009.09.008 19942021

[26] Wolfenden L , Kingsland M , Rowland BC , Dodds P , Gillham K , Yoong SL , Improving availability, promotion and purchase of fruit and vegetable and non–sugar-sweetened drink products at community sporting clubs: a randomised trial. Int J Behav Nutr Phys Act 2015;12(1):35. 10.1186/s12966-015-0193-5 25886467 PMC4396565

[27] Le HN Gold L , Abbott G , Crawford D , McNaughton SA , Mhurchu CN , Economic evaluation of price discounts and skill-building strategies on purchase and consumption of healthy food and beverages: the SHELf randomized controlled trial. Soc Sci Med 2016;159:83–91. 10.1016/j.socscimed.2016.04.015 27176465

[28] Ball K , McNaughton SA , Le HND , Gold L , Ni Mhurchu C , Abbott G , Influence of price discounts and skill-building strategies on purchase and consumption of healthy food and beverages: outcomes of the Supermarket Healthy Eating for Life randomized controlled trial. Am J Clin Nutr 2015;101(5):1055–64. 10.3945/ajcn.114.096735 25877492

[29] Olstad DL , Ball K , Abbott G , McNaughton SA , Le HN , Ni Mhurchu C , A process evaluation of the Supermarket Healthy Eating for Life (SHELf) randomized controlled trial. Int J Behav Nutr Phys Act 2016;13(1):27. 10.1186/s12966-016-0352-3 26912177 PMC4766691

[30] Ball K , McNaughton SA , Mhurchu CN , Andrianopoulos N , Inglis V , McNeilly B , Supermarket Healthy Eating for Life (SHELf): protocol of a randomised controlled trial promoting healthy food and beverage consumption through price reduction and skill-building strategies. BMC Public Health 2011;11(1):715. 10.1186/1471-2458-11-715 21936957 PMC3186753

[31] Ni Mhurchu C , Blakely T , Jiang Y , Eyles HC , Rodgers A . Effects of price discounts and tailored nutrition education on supermarket purchases: a randomized controlled trial. Am J Clin Nutr 2010;91(3):736–47. 10.3945/ajcn.2009.28742 20042528

[32] Blakely T , Ni Mhurchu C , Jiang Y , Matoe L , Funaki-Tahifote M , Eyles HC , Do effects of price discounts and nutrition education on food purchases vary by ethnicity, income and education? Results from a randomised, controlled trial. J Epidemiol Community Health 2011;65(10):902–8. 10.1136/jech.2010.118588 21296903

[33] Mhurchu CN , Blakely T , Funaki-Tahifote M , McKerchar C , Wilton J , Chua S , Inclusion of indigenous and ethnic minority populations in intervention trials: challenges and strategies in a New Zealand supermarket study. J Epidemiol Community Health 2009;63(10):850–5. 10.1136/jech.2008.081109 19574245

[34] Ni Mhurchu C , Blakely T , Wall J , Rodgers A , Jiang Y , Wilton J . Strategies to promote healthier food purchases: a pilot supermarket intervention study. Public Health Nutr 2007;10(6):608–15. 10.1017/S136898000735249X 17381930

[35] Cárdenas MK , Benziger CP , Pillay TD , Miranda JJ . The effect of changes in visibility and price on fruit purchasing at a university cafeteria in Lima, Peru. Public Health Nutr 2015;18(15):2742–9. 10.1017/S1368980014002730 25434293 PMC5454487

[36] Lindsay S , Lambert J , Penn T , Hedges S , Ortwine K , Mei A , Monetary matched incentives to encourage the purchase of fresh fruits and vegetables at farmers markets in underserved communities. Prev Chronic Dis 2013;10:E188. 10.5888/pcd10.130124 24229571 PMC3830923

[37] Anderson JV , Bybee DI , Brown RM , McLean DF , Garcia EM , Breer ML , 5 A Day fruit and vegetable intervention improves consumption in a low income population. J Am Diet Assoc 2001;101(2):195–202. 10.1016/S0002-8223(01)00052-9 11271692

[38] Herman DR , Harrison GG , Afifi AA , Jenks E . Effect of a targeted subsidy on intake of fruits and vegetables among low-income women in the Special Supplemental Nutrition Program for Women, Infants, and Children. Am J Public Health 2008;98(1):98–105. 10.2105/AJPH.2005.079418 18048803 PMC2156076

[39] Herman DR , Harrison GG , Jenks E . Choices made by low-income women provided with an economic supplement for fresh fruit and vegetable purchase. J Am Diet Assoc 2006;106(5):740–4. 10.1016/j.jada.2006.02.004 16647335

[40] Freedman DA , Mattison-Faye A , Alia K , Guest MA , Hébert JR . Comparing farmers’ market revenue trends before and after the implementation of a monetary incentive for recipients of food assistance. Prev Chronic Dis 2014;11:E87. 10.5888/pcd11.130347 24854238 PMC4032058

[41] Bihan H , Méjean C , Castetbon K , Faure H , Ducros V , Sedeaud A , Impact of fruit and vegetable vouchers and dietary advice on fruit and vegetable intake in a low-income population. Eur J Clin Nutr 2012;66(3):369–75. 10.1038/ejcn.2011.173 21989324

[42] Bihan H , Castetbon K , Mejean C , Peneau S , Pelabon L , Jellouli F , Sociodemographic factors and attitudes toward food affordability and health are associated with fruit and vegetable consumption in a low-income French population. J Nutr 2010;140(4):823–30. 10.3945/jn.109.118273 20181785

[43] Weinstein E , Galindo RJ , Fried M , Rucker L , Davis NJ . Impact of a focused nutrition educational intervention coupled with improved access to fresh produce on purchasing behavior and consumption of fruits and vegetables in overweight patients with diabetes mellitus. Diabetes Educ 2014;40(1):100–6. 10.1177/0145721713508823 24159007

[44] Smith C , Parnell WR , Brown RC , Gray AR . Providing additional money to food-insecure households and its effect on food expenditure: a randomized controlled trial. Public Health Nutr 2013;16(8):1507–15. 10.1017/S1368980012003680 22877571 PMC10271462

[45] French SA , Story M , Fulkerson JA , Hannan P . An environmental intervention to promote lower-fat food choices in secondary schools: outcomes of the TACOS Study. Am J Public Health 2004;94(9):1507–12. 10.2105/AJPH.94.9.1507 15333303 PMC1448482

[46] French SA , Story M , Fulkerson JA , Gerlach AF . Food environment in secondary schools: a la carte, vending machines, and food policies and practices. Am J Public Health 2003;93(7):1161–7. 10.2105/AJPH.93.7.1161 12835203 PMC1447927

[47] Fulkerson JA , French SA , Story M , Nelson H , Hannan PJ . Promotions to increase lower-fat food choices among students in secondary schools: description and outcomes of TACOS (Trying Alternative Cafeteria Options in Schools). Public Health Nutr 2004;7(5):665–74. 10.1079/PHN2003594 15251057

[48] Neumark-Sztainer D , French SA , Hannan PJ , Story M , Fulkerson JA . School lunch and snacking patterns among high school students: associations with school food environment and policies. Int J Behav Nutr Phys Act 2005;2(1):14. 10.1186/1479-5868-2-14 16209716 PMC1266392

[49] Shannon C , Story M , Fulkerson JA , French SA . Factors in the school cafeteria influencing food choices by high school students. J Sch Health 2002;72(6):229–34. 10.1111/j.1746-1561.2002.tb07335.x 12212407

[50] Hamdan S , Story M , French SA , Fulkerson JA , Nelson H . Perceptions of adolescents involved in promoting lower-fat foods in schools: associations with level of involvement. J Am Diet Assoc 2005;105(2):247–51. 10.1016/j.jada.2004.11.030 15668683

[51] Burr ML , Trembeth J , Jones KB , Geen J , Lynch LA , Roberts ZE . The effects of dietary advice and vouchers on the intake of fruit and fruit juice by pregnant women in a deprived area: a controlled trial. Public Health Nutr 2007;10(6):559–65. 10.1017/S1368980007249730 17381912

[52] Stites SD , Singletary SB , Menasha A , Cooblall C , Hantula D , Axelrod S , Pre-ordering lunch at work. Results of the What to Eat for Lunch study. Appetite 2015;84:88–97. 10.1016/j.appet.2014.10.005 25308434

[53] Thorndike AN , Riis J , Levy DE . Social norms and financial incentives to promote employees’ healthy food choices: a randomized controlled trial. Prev Med 2016;86:12–8. 10.1016/j.ypmed.2016.01.017 26827617 PMC4837037

[54] An R , Patel D , Segal D , Sturm R . Eating better for less: a national discount program for healthy food purchases in South Africa. Am J Health Behav 2013;37(1):56–61. 10.5993/AJHB.37.1.6 22943101 PMC3433851

[55] Sturm R , An R , Segal D , Patel D . A cash-back rebate program for healthy food purchases in South Africa: results from scanner data. Am J Prev Med 2013;44(6):567–72. 10.1016/j.amepre.2013.02.011 23683973 PMC3659342

[56] Olsho LEW , Klerman JA , Wilde PE , Bartlett S . Financial incentives increase fruit and vegetable intake among Supplemental Nutrition Assistance Program participants: a randomized controlled trial of the USDA Healthy Incentives Pilot. Am J Clin Nutr 2016;104(2):423–35. 10.3945/ajcn.115.129320 27334234

[57] An R . Nationwide expansion of a financial incentive program on fruit and vegetable purchases among Supplemental Nutrition Assistance Program participants: a cost-effectiveness analysis. Soc Sci Med 2015;147:80–8. 10.1016/j.socscimed.2015.09.032 26547363

[58] Kral TVE , Bannon AL , Moore RH . Effects of financial incentives for the purchase of healthy groceries on dietary intake and weight outcomes among older adults: a randomized pilot study. Appetite 2016;100:110–7. 10.1016/j.appet.2016.02.022 26879224 PMC4799756

[59] Phipps EJ , Braitman LE , Stites SD , Singletary SB , Wallace SL , Hunt L , Impact of a rewards-based incentive program on promoting fruit and vegetable purchases. Am J Public Health 2015;105(1):166–72. 10.2105/AJPH.2013.301752 24625144 PMC4265942

[60] Phipps EJ , Kumanyika SK , Stites SD , Singletary SB , Cooblall C , DiSantis KI . Buying food on sale: a mixed methods study with shoppers at an urban supermarket, Philadelphia, Pennsylvania, 2010–2012. Prev Chronic Dis 2014;11:E151. 10.5888/pcd11.140174 25188276 PMC4157594

[61] Phipps EJ , Wallace SL , Stites SD , Uplinger N , Brook Singletary S , Hunt L , Using rewards-based incentives to increase purchase of fruit and vegetables in lower-income households: design and start-up of a randomized trial. Public Health Nutr 2013;16(5):936–41. 10.1017/S1368980012004934 23168307 PMC10271350

[62] Falbe J , Thompson HR , Becker CM , Rojas N , McCulloch CE , Madsen KA . Impact of the Berkeley excise tax on sugar-sweetened beverage consumption. Am J Public Health 2016;106(10):1865–71. 10.2105/AJPH.2016.303362 27552267 PMC5024386

[63] Falbe J , Rojas N , Grummon AH , Madsen KA . Higher retail prices of sugar-sweetened beverages 3 months after Implementation of an Excise Tax in Berkeley, California. Am J Public Health 2015;105(11):2194–201. 10.2105/AJPH.2015.302881 26444622 PMC4605188

[64] Jensen JD , Smed S . The Danish tax on saturated fat — short run effects on consumption, substitution patterns and consumer prices of fats. Food Policy 2013;42:18–31. 10.1016/j.foodpol.2013.06.004

[65] Smed S , Scarborough P , Rayner M , Jensen JD . The effects of the Danish saturated fat tax on food and nutrient intake and modelled health outcomes: an econometric and comparative risk assessment evaluation. Eur J Clin Nutr 2016;70(6):681–6. 10.1038/ejcn.2016.6 27071513

[66] Jensen JD , Smed S , Aarup L , Nielsen E . Effects of the Danish saturated fat tax on the demand for meat and dairy products. Public Health Nutr 2015;19(17):1–10. 26306542 10.1017/S1368980015002360 PMC10270788

[67] Juhl HJ , Jensen MB . Relative price changes as a tool to stimulate more healthy food choices — a Danish household panel study. Food Policy 2014;46:178–82. 10.1016/j.foodpol.2014.03.008

[68] Batis C , Rivera JA , Popkin BM , Taillie LS . First-year evaluation of Mexico’s tax on nonessential energy-dense foods: an observational study. PLoS Med 2016;13(7):e1002057. 10.1371/journal.pmed.1002057 27379797 PMC4933356

[69] Colchero MA , Popkin BM , Rivera JA , Ng SW . Beverage purchases from stores in Mexico under the excise tax on sugar sweetened beverages: observational study. BMJ 2016;352:h6704. 10.1136/bmj.h6704 26738745 PMC4986313

[70] Colchero MA , Salgado JC , Unar-Munguía M , Hernández-Ávila M , Rivera-Dommarco JA . Price elasticity of the demand for sugar sweetened beverages and soft drinks in Mexico. Econ Hum Biol 2015;19:129–37. 10.1016/j.ehb.2015.08.007 26386463

[71] Colchero MA , Salgado JC , Unar-Munguía M , Molina M , Ng S , Rivera-Dommarco JA . Changes in prices after an excise tax to sweetened sugar beverages was implemented in Mexico: evidence from urban areas. PLoS One 2015;10(12):e0144408. 10.1371/journal.pone.0144408 26675166 PMC4682930

[72] Darmon N , Lacroix A , Muller L , Ruffieux B . Food price policies improve diet quality while increasing socioeconomic inequalities in nutrition. Int J Behav Nutr Phys Act 2014;11(66):66. 10.1186/1479-5868-11-66 24886414 PMC4045909

[73] Harnack L , Oakes JM , Elbel B , Beatty T , Rydell S , French S . Effects of subsidies and prohibitions on nutrition in a food benefit program: a randomized clinical trial. JAMA Intern Med 2016;176(11):1610–8. 10.1001/jamainternmed.2016.5633 27653735 PMC5988257

[74] Deliens T , Deforche B , Annemans L , De Bourdeaudhuij I , Clarys P . Effectiveness of pricing strategies on French fries and fruit purchases among university students: results from an on-campus restaurant experiment. PLoS One 2016;11(11):e0165298. 10.1371/journal.pone.0165298 27812123 PMC5094693

[75] Steckler A , Linnan L . Process evaluation for public health interventions and research. San Francisco (CA): Jossey-Bass; 2002.

[76] Niebylski ML , Redburn KA , Duhaney T , Campbell NR . Healthy food subsidies and unhealthy food taxation: a systematic review of the evidence. Nutrition 2015;31(6):787–95. 10.1016/j.nut.2014.12.010 25933484

[77] An RP . Effectiveness of subsidies in promoting healthy food purchases and consumption: a review of field experiments. Public Health Nutr 2014;17(8):1215–28. 10.1017/S1368980013002024 PMC3898771 23122423

[78] Chung J , Li D . The prospective impact of a multi-period pricing strategy on consumer perceptions for perishable foods. Br Food J 2013;115(3):377–93. 10.1108/00070701311314200

[79] Ledoux TA , Hingle MD , Baranowski T . Relationship of fruit and vegetable intake with adiposity: a systematic review. Obes Rev 2011;12(5):e143–50. 10.1111/j.1467-789X.2010.00786.x 20633234

[80] Waterlander WE , de Mul A , Schuit AJ , Seidell JC , Steenhuis IHM . Perceptions on the use of pricing strategies to stimulate healthy eating among residents of deprived neighbourhoods: a focus group study. Int J Behav Nutr Phys Act 2010;7(1):44. 10.1186/1479-5868-7-44 20482857 PMC2885313

[81] Langellier BA , Garza JR , Prelip ML , Glik D , Brookmeyer R , Ortega AN . Corner store inventories, purchases, and strategies for intervention: a review of the literature. Calif J Health Promot 2013;11(3):1–13. 25374481 PMC4217697

[82] Story M , Lytle LA , Birnbaum AS , Perry CL . Peer-led, school-based nutrition education for young adolescents: feasibility and process evaluation of the TEENS study. J Sch Health 2002;72(3):121–7. 10.1111/j.1746-1561.2002.tb06529.x 11962228

[83] Budd N , Jeffries JK , Jones-Smith J , Kharmats A , McDermott AY , Gittelsohn J . Store-directed price promotions and communications strategies improve healthier food supply and demand: impact results from a randomized controlled, Baltimore City store-intervention trial. Public Health Nutr 2017; Epub ahead of print. 10.1017/S1368980017000064 28222818 PMC5725746

