# Supplementary material for: Pricing Strategies to Encourage Availability, Purchase, and Consumption of Healthy Foods and Beverages: A Systematic Review
Source: Prev Chronic Dis. 2017 Nov 2;14:E107. doi: 10.5888/pcd14.170213 (PMC5672888; doi:10.5888/pcd14.170213)
Supplement: Supplementary file 1 [file 17_0213Appendix.docx]

**Appendix. Pricing Strategies to Encourage Availability, Purchase, and Consumption of Healthy Foods and Beverages: A Systematic Review**

**1) MEDLINE MeSH terms – return: 256 studies**

(((“Consumer behavior” [Mesh] OR “Consumer Behavior” [tiab] OR “Marketing” [Mesh] OR “Marketing” [tiab] OR “Cost Savings” [Mesh] OR “Cost Savings” [tiab] OR “Health Promotion” [Mesh] OR “Health Promotion” [tiab] OR “Food Supply” [Mesh] OR “Food Supply” [tiab])))

AND

((“Diet” [Mesh] OR “Diet” [tiab] OR “Eating” [Mesh] OR “Food Intake” [tiab] OR “Eating” [tiab] OR “dietary behavior” OR “dietary intake” OR “eating behavior” OR “Food Intake” [Mesh] OR “Diet, Food, and Nutrition” [Mesh] OR “Diet, Food, and Nutrition” [tiab] OR “Snacks” [Mesh] OR “Snacks” [tiab] OR “Food and Beverages” [Mesh] OR “Food and Beverages” [tiab]))

AND

((“Incentive Reimbursement” [Mesh] OR “Incentive Reimbursement” [tiab] OR “Incentive Reimbursement” OR “Taxes” [Mesh] OR “Taxes” [tiab] “Economics” [Mesh]” OR “Financial Support” [Mesh] OR “Financial Support” [tiab] “Disincentive” [Mesh] OR “Disincentive” [tiab] OR “Disincentive” OR “Motivation” [Mesh] OR “Motivation” [tiab] OR “Reward” [Mesh] OR “Reward” [tiab] “Commerce” [Mesh] OR “Pricing Strategies” [tiab] OR Pricing Strategies))

**2) Embase search: return 163**

'diet':ab,ti OR 'eating':ab,ti OR 'food intake':ab,ti OR 'diet, food, and nutrition':ab,ti OR 'snacks':ab,ti OR 'food and beverages':ab,ti OR 'dietary behavior':ab,ti OR 'dietary intake':ab,ti OR 'eating behavior':ab,ti

AND

'consumer behavior':ab,ti OR 'marketing':ab,ti OR 'cost savings':ab,ti OR 'health promotion':ab,ti OR 'food supply':ab,ti OR 'food purchasing':ab,ti

AND

'incentive reimbursements':ab,ti OR 'taxes':ab,ti OR 'economics':ab,ti OR 'financial support':ab,ti OR 'disincentive':ab,ti OR 'motivation':ab,ti OR 'reward':ab,ti OR 'commerce':ab,ti OR 'pricing strategies':ab,ti OR 'monetary incentive':ab,ti OR 'monetary disincentive':ab,ti OR 'financial disincentive':ab,ti

Refine:

AND ([controlled clinical trial]/lim OR [randomized controlled trial]/lim) AND [article]/lim AND [humans]/lim AND [english]/lim AND [abstracts]/lim AND [embase]/lim AND [2000–2016]/py

**3) Web of Science – return: 237 studies**

TS= (pricing strategy OR incentives AND (food OR consumer behavior OR diet)) 17,612

TI= (pricing strategy AND (food OR consumer behavior OR diet))) AND LANGUAGE: (English) AND DOCUMENT TYPES: (Article) 16

i) TI=(food OR consumer behavior OR diet)) AND LANGUAGE: (English) AND DOCUMENT TYPES: (Article) AND TS= (monetary incentive OR disincentive OR taxes)) *AND* **LANGUAGE:** (English) *AND* **DOCUMENT TYPES:** (Article)

ii) TI= (pricing strategy AND (food OR consumer behavior OR diet))) AND LANGUAGE: (English) AND DOCUMENT TYPES: (Article)

i OR ii = 235

**4) PsychINFO – return: 775**

(KW (food OR consumer behavior OR diet) AND (KW (pricing strategies OR taxes Or incentives OR disincentive) )

Refined: by year, language, peer–reviewed, human only

**5) Cochrane – return: 584**

(('incentive reimbursements':kw or 'taxes':kw or 'economics':kw or 'financial support':kw or 'disincentive':kw or 'motivation':kw or 'reward':kw or 'commerce':kw or 'pricing strategies':kw or 'monetary incentive':kw or 'monetary disincentive':kw or 'financial disincentive':kw)

AND

('diet':ti or 'eating':ti or 'food intake':ti or 'diet, food, and nutrition':ab,ti or 'snacks':ti or 'food and beverages':ti or 'dietary behavior':ti or 'dietary intake':ti or 'eating behavior':ti)

OR

('consumer behavior':ti or 'marketing':ab,ti or 'cost savings':ab,ti or 'health promotion':ab,ti or 'food supply':ab,ti or 'food purchasing':ab,ti))

**6) Clinicaltrials.gov – return: 61**

("pricing incentive" OR "pricing strategy" OR "taxes") AND ("food" OR "nutrition" OR "diet" OR "food purchase")

Appendix Table 1: Definitions of Review Criteria

| **Review criteria** | **Definition** |
| --- | --- |
| Project name | We have included the intervention program/study name where one was given. When one was not provided, we described the study as “Not named” and provided a brief descriptive title based on our understanding of the study. |
| Study design | Study design was reported by the authors. When one was not reported, we specified the study design based on our understanding of the study. All study arms are specified in this section. |
| Sample size | We specified the number of intervention venues, study participants, and/or sales records that was reported in each study, where relevant. When the recruited sample size and the analytic sample size differed, we considered the recruited sample as the final sample size. |
| Study duration | Study duration was specified by the authors. We calculated the total intervention duration, excluding baseline and follow–up assessment periods. |
| Target population | We described the characteristics of the group who received the program/intervention. |
| Model/theory | We included an underlying framework of each study that was used to develop its intervention components, if any was mentioned by the authors. |
| Goal or purpose of the trial | Study investigators generally provided an overall goal or purpose of the intervention. When one was not provided, we described the purpose based on our understanding of the study. |
| Food/beverages that were the intervention’s focus | We identified the specific foods or beverages that the study aimed to promote or de–promote. |
| Food/beverage sources | We specified intervention venues described by the authors. We also included the number of intervention venues if it was provided in the publication. |
| Intervention strategies: pricing or cost | We described the intervention strategies that target price in some fashion, including incentives (coupons, vouchers, discounts, rebates), and disincentives (price increase, taxes). |
| Intervention strategies: changing availability of healthy and unhealthy foods | We included any intervention component that increased or decreased the availability of healthy and unhealthy foods. |
| Intervention strategies: changing location of healthy/unhealthy foods | We included a description of any intervention component that relocated healthy and unhealthy foods. |
| Intervention strategies: labelling of healthy and unhealthy foods | We included any intervention component that changed labeling of healthy and unhealthy food. This includes shelf labels, signage, and posters that aimed to promote or de–promote healthy and unhealthy products. |
| Intervention strategies: policy (eg, taxes) | This characteristic was described if the pricing intervention component included policy–level changes, such as taxes. |
| Other approaches | All other approaches that were not one of the intervention components mentioned above were included here. These include nutrition education or training of staff, for example. |
| Formative research | We identified all information–gathering activities that were conducted to inform intervention development. Formative research was conducted before the implementation, and could include both qualitative and quantitative approaches. |
| Feasibility assessment (acceptability, operability, perceived sustainability) | We examined if the study collected data on economic or cultural acceptability, operability, and/or perceived sustainability of the intervention. |
| Process evaluation (how well the program was implemented according to plan) | We included all activities during the study that assessed how well an intervention was implemented according to study plan, with attention paid to the use of the constructs of reach, dose delivered, and fidelity |
| Impact measures: retail level | We identified how each study measured impact of its intervention at retail–level (ie, stocking, sales). Retail–level measures do not include individual–level assessments such as changes in purchasing or eating behavior. |
| Impact measures: consumer level (psychosocial, behavioral, health outcomes) | We identified how each study measured impact of its intervention at the individual level. Specifically, we examined psychosocial, behavioral, and health assessments at the consumer level. |
| Feasibility and process results | We showed the results of feasibility and process evaluation. The format on how we reported the findings followed what was examined in feasibility and process evaluation sections. |
| Impact results: retail stocking and sales | We report the results of intervention impact at the retail level, including changes in stocking and sales. |
| Impact results: consumer psychosocial measures | We reported findings on psychosocial assessment of the intervention at the consumer level, including intentions and attitudes toward intervention components. |
| Impact results: consumer behavioral measures | We reported findings on behavioral assessment, such as changes in food purchasing and consumption. |
| Impact results: consumer health outcomes | We reported findings on study participants’ health outcomes. |
| Sustainability | Study assessment of maintenance of behavior or food availability after the implementation of the intervention through follow–up measurement (after 6 months of the intervention) and/or qualitative assessment. Report of continuity of the study by third parties was also considered sustainability. |
| Quality of the research | We used criteria described by An R, 2013 (9). The quality score assessed the presence or absence of ten dichotomous criteria, as follows: 1) a control group was included; 2) baseline characteristics between the control and intervention groups were similar; 3) the intervention period was at least 5 weeks; 4) the follow–up period was at least 3 weeks; 5) an objective measure of food purchases or intake was used; 6) the measurement tool was shown to be reliable and valid in previously published studies; 7) participants were randomly recruited with a response rate of 60% or higher; 8) attrition was analyzed and determined not to differ significantly by respondents’ baseline characteristics between the control and intervention groups; 9) potential confounders were properly controlled for in the analysis; and 10) intervention procedures were documented in detail in the article. A total study quality score ranging from 0 to 10 was obtained for each study by summing up these criteria. Two reviewers assessed quality of each study independently, and final score was adjudicated by the first author. |
| Limitations | We included limitations of the study that were reported in the publications. |
| Study recommendations | Study recommendations that were provided by the authors were included. If no recommendation was provided, we reported “none”. |

Appendix Table 2. Formative research, feasibility and process evaluation strategies and results of studies

| Study Name | Formative Research | Feasibility | Process Evaluation | Feasibility Results | Process results |
| --- | --- | --- | --- | --- | --- |
| **Financial discounts on healthier food and beverages** | | | | | |
| Baltimore Healthy Carryouts (10–17) | +++  In–depth interviews, focus groups, conjoint analysis, Ground–truthing, direct observation. Intervention materials designed with feedback from the community | + (Acceptability) | +++  (Reach, dose received, fidelity) | High acceptability, especially the new menu boards shown to be feasible. | Moderate to high dose received, high fidelity and reach |
| B’More Healthy Retail Rewards (18,19) | +++  In–depth interviews, observations, and focus groups with small store owners and consumers. | +  (Feasibility) | +++  (Reach, dose received, fidelity) | High feasibility  Combined intervention showed greater discount (Phase 2) | NA |
| Not named (healthy foods at swimming pools) (20) | ++  Developed and pre–tested observation forms and descriptive names for healthy items | +  (Operability) | +  (Fidelity) | High acceptability  Children interacted with the display and taste test  Challenges offering healthy menu items | NA |
| HealthWorks (21–24) | +  Worksite food inventory | +  (Employed Advisory panel) | +  (Fidelity) | Low feasibility, pricing intervention was not implemented at any site | Moderate fidelity |
| Not named (Mississippi Healthy Beverages) (25) | – | – | – | High acceptability | NA |
| Not named (multi–component intervention in sports clubs) (26) | +  Pilot test of the survey questionnaire | + (Sustainability) | +  (Fidelity)  Financial records of canteen revenues | High feasibility  Intervention clubs offered meal deals and reduced price of promoted fruits and vegetables | NA |
| Supermarket Healthy Eating for Life (SHELf) trial (27–30) | +  Pilot test of Skill–building materials. | +  (Sustainability) | ++  (Reach, dose received) | High sustainability | NA |
| Supermarket Healthy Options Project (SHOP) (31–34) | +++  6 Focus groups for intervention planning; Pilot of barcode scanning terminals | +  (Operability) | – | Moderate acceptability Low sustainability | Moderate reach, dose received, fidelity |
| Not named (Lima University cafeteria study) (35) | – | +  (Acceptability) | +  (Fidelity) | High feasibility | NA |
| **Redeemable coupons/vouchers for healthier foods and beverages targeting participants in food assistant programs** | | | | | |
| Farmers Market Fresh Fund Incentive Program (36) | – | +  (Perceived sustainability) | – | NA | NA |
| Project FRESH (Farm Resources Encouraging and Supporting Health) (37) | +  Two focus groups conducted to develop questionnaire | – | +  (Dose received) | High awareness | NA |
| Not named (Los Angeles economic subsidy) (38,39) | – | +  (Operability) | +  (Fidelity) | High feasibility | NA |
| Shop N Save (40) | – | – | – | NA | NA |
| **Redeemable coupons/vouchers for healthier foods and beverages targeting nonparticipants in food assistant programs** | | | | | |
| Not named (French supermarkets) (41,42) | – | ++ (Acceptability, feasibility) assessed at 3 months | – | Moderate feasibility | NA |
| Not named (New York City farmers markets) (43) | – | – | – | High feasibility | NA |
| Spend Study (44) | – | – | – | NA | NA |
| Trying Alternative Cafeteria Options in Schools (TACOS) (45–50) | +++  Environmental assessment, collected information on food inventory, school food policies, practices, lunch patterns | +  (Operability) | ++  (Dose received, fidelity) | NA | High fidelity, dose received |
| Not named (United Kingdom fruit juice delivery) (51) | – | – | – | Moderate feasibility | NA |
| What to Eat for Lunch study (52) | – | +++ | – | High sustainability | NA |
| **Cash–back rebate** | | | | | |
| Not named (Boston social norm and rebate study) (53) | +  Pilot test of an intervention material | + (Sustainability) | – | NA | NA |
| Healthy Food program (54,55) | – | – | – | NA | NA |
| Healthy Incentives Pilot (HIP) (56,57) | +  Pre–tested survey instruments | +  (Operability) | +  (Dose received) | High feasibility–Dose received: HIP exposure was higher than non–HIP–Awareness: Low–Moderate | High dose received in intervention group; Low – moderate awareness |
| Not named (Philadelphia financial incentives) (58) | – | – | +  (Dose received) | NA | NA |
| Rewards study (59–61) | ++  12–week pilot study that informed the design, incentive method, and eligibility criteria for the study | +++  (Feasibility, Operability, Sustainability) | – | High acceptability and operability | NA |
| **Disincentives for unhealthy foods and beverages, with and without incentives for healthy foods and beverage purchases** | | | | | |
| Berkeley, California, excise tax on soda (62,63) | – | +  (Operability) | – | NA | NA |
| Danish saturated fat tax (64–66,77) | – | +  (Operability) | – | Moderate feasibility | NA |
| Excise tax on SSBs in Mexico (67–70) | – | – | +  (Fidelity) | High feasibility | NA |
| Not named (French food baskets) (71) | – | – | – | NA | NA |
| Not named (Minneapolis financial incentives) (72) | – | +  (Operability) | – | NA | NA |
| Not named (Brussels University cafeteria study) (73) | +  Exploratory analysis of dietary intakes of students | ++ (Acceptability, operability) | – | Higher acceptability of price reduction on healthy products than in price increase unhealthy products | NA |

Note: + indicates that the assessments of interests were conducted, whereas – indicates no assessment of interests was mentioned. Number of + indicates the number of assessments that were conducted. NA means not assessed.
